# Generalized arteriomegaly as a precursor to multifocal aneurysmal disease: implications for early intervention

**DOI:** 10.3389/fcell.2025.1592225

**Published:** 2025-05-20

**Authors:** Zhaohua Cai, Ben He

**Affiliations:** Department of Cardiology, Shanghai Chest Hospital, Shanghai Jiao Tong University School of Medicine, Shanghai, China

**Keywords:** abdominal aortic aneurysm, arteriomegaly, dilation, LKB1 (STK11), mouse model

## 1 Introduction

Arteriomegaly refers to a diffuse arterial ectasia, which may manifest independently or concomitantly with aneurysmal pathology. This complex vasculopathy is characterized by pronounced dilation, excessive tortuosity, segmental elongation, and luminal irregularities accompanied by hemodynamic flow deceleration. While initially conceptualized by Leriche in 1942 using the descriptive term “*dolicho et mega artere*” (denoting elongated and enlarged arteries) (R, 1942), the entity remained poorly characterized until Thomas described the detailed angiographic findings and established the current nomenclature arteriomegaly in 1971 (Thomas, 1971). Pathoanatomically, arteriomegaly diverges from classical aneurysms, which are strictly defined as focal, persistent dilatations exceeding 1.5 times the reference arterial diameter. Emerging evidence reveals significant pathophysiological correlations between arteriomegaly and aneurysm development, particularly in the abdominal aorta and peripheral arterial systems, suggesting shared mechanisms underlying both pathological processes.

Clinical investigations have increasingly demonstrated the coexistence of systemic arteriomegaly in abdominal aortic aneurysm (AAA) pathogenesis. Tilson et al. initially documented concurrent suprarenal aortic and iliac arterial dilatation in AAA cohorts (Tilson and Dang, 1981), a finding later expanded by Baxter et al. who identified pan-aortic dilation (encompassing ascending/descending thoracic, supraceliac, and suprarenal segments) in patients with infrarenal AAA (Baxter et al., 1994). Notably, the arterial remodeling extends beyond the aortic territory in AAA patients. Comparative ultrasonographic analyses by Ward et al. revealed significant diameter increases in carotid, brachial, femoral, and popliteal arteries among AAA patients versus controls (Ward, 1992). This pattern of peripheral vascular involvement is further corroborated by multiple studies demonstrating statistically significant common carotid enlargement in AAA patients (Makita et al., 2000; Iwamoto et al., 2004; van Laake et al., 2005; Nordon et al., 2009). Furthermore, generalized arteriomegaly frequently coexists with multisegmental aneurysms in popliteal artery aneurysm cases (Yamamoto et al., 2002; Widmer et al., 2008). These multisystem manifestations collectively redefine aneurysmal disease as a pan-vascular disorder rather than localized pathology.

The progressive nature of arteriomegaly is underscored by its strong temporal association with multifocal aneurysm development (Thomas, 1971; Carlson et al., 1975; Hollier et al., 1983; Chan and Thomas, 1990; Barandiaran et al., 2012). Current epidemiological data reveal that 30.3%–80.6% of arteriomegaly patients develop multiple or diffuse aneurysms in arteriomegalic populations (Table 1), with longitudinal studies demonstrating critical progression patterns (Thomas, 1971; Carlson et al., 1975; Hollier et al., 1983; Chan and Thomas, 1990; Barandiaran et al., 2012). A pivotal 76-month cohort study documented baseline aneurysm prevalence of 53.7% at diagnosis, escalating to 80.6% during surveillance through *de novo* formation across distinct arterial territories. This characteristic pattern of temporal-spatial progression—marked by sequential aneurysm development in previously unaffected vascular regions—provides compelling evidence for redefining arteriomegaly as a progressive pan-arteriopathy (Barandiaran et al., 2012). Such insights fundamentally alter therapeutic paradigms, necessitating a shift from localized, lesion-specific interventions to systemic strategies targeting the underlying pan-vascular pathophysiology.

**TABLE 1:** Demographic characteristics and aneurysm incidence in published cases of patients with arteriomegaly.

Author, year	N^a^	Age	Aneurysm	Multiple/Diffuse aneurysms
Thomas (Thomas, 1971), 1971	30	Range 46–75 years (mean 66 years)	20/30 (66.6%)	11/30 (36.7%) (multiple)
Carlson (Carlson et al., 1975), 1975	7	Range 64–89 years (mean 71 years)	4/7 (57%)	4/7 (57%) (multiple)
Hollier (Hollier et al., 1983), 1983	300	Range 36.5–87 years (mean 67.5 years)	—	91/300 (30.3%) (diffuse)
Chan (Chan and Thomas, 1990), 1990	65	Range 36–86 years (mean 66 years)	53/65 (81.5%)	21/65 (32.3%) (multiple)
Barandiaran (Barandiaran et al., 2012), 2012	67	Range 60–89 years (mean 74 years)	—	36/67 (53.7%) (at presentation) 54/67 (80.6%) (76 months follow-up)

^a^ Patients with arteriomegaly

Nevertheless, the precise etiopathogenetic mechanisms governing the co-evolution of generalized arteriomegaly and focal aneurysm formation remain elusive, constrained by two research limitations. First, the current experimental paradigm suffers from a critical absence of genetically faithful animal models spontaneously recapitulating the full spectrum of human arteriomegaly-aneurysm progression. Second, human translational studies are fundamentally limited to histopathological analysis of end-stage surgical specimens, which by their terminal nature inherently obscure inciting molecular events. This dual barrier impedes delineation of molecular drivers underlying macroscopic vascular changes, underscoring the urgent need for preclinical models that recapitulate generalized arteriomegaly and aneurysm co-evolution.

## 2 Discovery of the murine model of generalized arteriomegaly and aortic/arterial aneurysm formation

Our laboratory engineered a tamoxifen-inducible smooth muscle cell (SMC)-specific Lkb1 knockout mouse model through *Myh11-CreER* ^*T2*^-mediated recombination in *Lkb1* ^*flox/flox*^ mice (Cai et al., 2024). Remarkably, *Lkb1* ^*flox/flox*^;*Myh11-CreER* ^*T2*^ mice invariably succumbed to fatal aortic/arterial rupture within 8 months post-tamoxifen induction (Cai et al., 2024). Serial *in vivo* micro-ultrasound imaging over 4.5 months demonstrated progressive lumen expansion in ascending aorta, abdominal aorta, and carotid arteries in *Lkb1* ^*flox/flox*^;*Myh11-CreER* ^*T2*^ mice versus control mice, establishing this model as manifesting generalized arteriomegaly (Cai et al., 2024). Systematic histomorphometric analysis of aortic segments and peripheral arteries in *Lkb1* ^*flox/flox*^;*Myh11-CreER* ^*T2*^ mice revealed concordant pathological dilation patterns. Specifically, hematoxylin-eosin staining quantitatively confirmed progressive luminal enlargement in carotid arteries, femoral arteries, and aortic regions, consistent precisely with ultrasound metrics (Cai et al., 2024). Notably, as time developed, *Lkb1* ^*flox/flox*^;*Myh11-CreER* ^*T2*^ mice exhibited aneurysm formation in abdominal aorta, renal artery, iliac artery, femoral artery, and/or popliteal artery leading to arterial rupture (Cai et al., 2024), which is highly reminiscent of human aneurysm of the aorta-iliac-femoral tree (Norman and Powell, 2010).

In our subsequent multimodal imaging investigation, we implemented longitudinal computed tomography angiography (CTA) and high-resolution magnetic resonance imaging (MRI) to delineate the spatiotemporal progression of aneurysmal disease in the *Lkb1* ^*flox/flox*^;*Myh11-CreER* ^*T2*^ model (Cai et al., 2025). Quantitative imaging protocols revealed several distinct pathological phases in *Lkb1* ^*flox/flox*^;*Myh11-CreER* ^*T2*^ mice: 1) systemic arteriomegaly with significant increases in cross-sectional diameters across aortic segments and multiple medium arteries (including left common carotid artery, celiac trunk, renal artery, common iliac artery, and femoral artery), 2) multiterritorial aneurysm development involving renal, iliac, caudal, and femoral arteries and abdominal aortas, and 3) terminal rupture events. This comprehensive phenotyping establishes that postnatal Lkb1 ablation in mature vascular smooth muscle cell (VSMC) induces a progressive vascular pathology: initial generalized arteriomegaly progressing to multifocal aneurysm formation, ultimately culminating in fatal rupture, recapitulating key aspects of human multiterritorial aneurysmal diseases. The anatomical distribution (aorto-iliac-femoral predominance) and temporal progression pattern (diffuse dilation preceding focal aneurysms) mirror clinical observations in aneurysmal patients.

Notably, conditional Lkb1 ablation in mature VSMC using the tamoxifen-inducible *Myh11-CreER* ^*T2*^ system initiated a sequential phenotypic transition characterized by progressive transdifferentiation. Initially, contractile VSMCs adopted an early modulated phenotype, followed by their transformation into fibroblast-like, chondrocyte-like (cartilage-forming), and ultimately osteocyte-like (calcification-prone) cells (Cai et al., 2024). Furthermore, Lkb1 deficiency triggered profound extracellular matrix (ECM) remodeling, including excessive deposition of collagen-rich and proteoglycan-rich matrix, as well as elastic lamina fragmentation and disruption (Cai et al., 2024). Collectively, these findings delineate a causal cascade wherein Lkb1 loss drives VSMC lineage reprogramming, which in turn precipitates ECM compositional and structural dysregulation. This multistep pathological cascade ultimately compromises vascular wall integrity, serving as a pivotal driver of arteriomegaly and aneurysm pathogenesis.

## 3 Clinical implications

### 3.1 Redefining arteriomegaly-aneurysm continuum

The *Lkb1* ^*flox/flox*^;*Myh11-CreER* ^*T2*^ murine model recapitulates a biphasic disease continuum: (1) an initial systemic pan-arterial ectasia phase marked by diffuse vascular dilatation, progressing to (2) focal aneurysm formation—a pathological trajectory that precisely mirrors the clinical progression from arteriomegaly to aneurysm formation in humans. While no existing murine models fully replicate this continuum, certain genetically engineered strains exhibit analogous vascular dysfunction (Quelquejay et al., 2024; Arévalo et al., 2023). Our findings resolve a longstanding nosological debate by demonstrating that generalized arteriomegaly and focal aneurysms represent either distinct phenotypic manifestations (systemic vs. localized) or sequential stages of a unified arteriopathy characterized by progressive loss of vascular wall integrity. This pathochronic relationship is corroborated by clinical epidemiology showing arteriomegaly typically manifests decades prior to aneurysmal presentation (Hollier et al., 1983). Importantly, longitudinal clinical studies by Barandiaran et al. revealed that 58% of arteriomegaly patients without baseline aneurysms developed multifocal lesions across the aorto-iliac-femoral axis during 76-month median follow-up (Barandiaran et al., 2012). These collective findings establish AAA as a representative manifestation of systemic arterial wall dysregulation rather than an isolated vascular pathology.

### 3.2 Mechanistic insight into aneurysm drivers

The *Lkb1* ^*flox/flox*^;*Myh11-CreER* ^*T2*^ murine model reveals an intrinsic cell-autonomous mechanism governing aneurysmal pathogenesis. Our findings establish that sequential VSMC transformation and maladaptive extracellular matrix (ECM) remodeling constitute the principal pathogenic drivers of generalized arteriomegaly and loss of vessel structural integrity. The initiation of VSMC transformation triggers ECM remodeling characterized by excessive collagen deposition, proteoglycan accumulation, and elastic lamina degradation, creating a feedforward loop that collectively compromises vascular wall integrity. The pathophysiological progression in *Lkb1* ^*flox/flox*^;*Myh11-CreER* ^*T2*^ model aligns with clinical observations of panvascular ECM abnormalities in AAA patients (Baxter et al., 1994; van Laake et al., 2005). While prior research emphasizes matrix metalloproteinase (MMP)-mediated proteolysis in the pathogenesis of aneurysm formation (Baxter et al., 1994), our findings position VSMC phenotypic determination as the upstream regulator initiating ECM dysregulation. This mechanistic paradigm reorients therapeutic strategies to preserving VSMC contractile identity, offering novel intervention opportunities during early disease progression.

### 3.3 Genetic contribution to aortic aneurysm

Despite the absence of definitively identified genetic loci in clinical cohorts to date, accumulating evidence underscores a pronounced familial predisposition to arteriomegaly and aneurysmal disease (Lawrence et al., 1998). These observations strongly suggest an underlying heritable component, even as the precise molecular drivers remain unresolved. Our recent study (Cai et al., 2024) provides mechanistic insights into this phenomenon, proposing that genetic mutations disrupting VSMC identity and homeostasis constitute a pivotal pathogenic axis in generalized arteriomegaly and aneurysm formation. Specifically, we hypothesize that VSMC transformation or “loss of fate” compromises arterial wall integrity, rendering vessels susceptible to pathological dilatation and aneurysm formation. This model aligns with the systemic nature of arteriomegaly, implicating pan-vascular defects in VSMC biology rather than localized environmental stressors. While candidate genes have yet to be validated in human AAA populations, our findings bridge clinical epidemiology (familial clustering) and cellular pathophysiology, advocating for a paradigm where inherited or *de novo* genetic alterations disrupt vascular homeostasis through impaired VSMC-dependent remodeling processes.

## 4 Conclusion and perspectives

Generalized arteriomegaly and localized aneurysm formation represent sequential pathological stages within a unified vascular disease continuum, both stemming from systemic dysregulation of arterial wall homeostasis. The tamoxifen-inducible *Lkb1* ^*flox/flox*^;*Myh11-CreER* ^*T2*^ murine model emerges as a superior preclinical platform for investigating this disease progression, as it faithfully recapitulates both the anatomical characteristics and pathophysiological mechanisms observed in human aneurysm formation. This genetically engineered model system provides unprecedented opportunities for early intervention strategy development through three unique capacities: 1) Spatiotemporal recapitulation of human disease progression—from diffuse arteriomegaly to aortic/iliac aneurysm rupture, 2) Identifying critical molecular pathways governing VSMC cell fate and vascular matrix remodeling, and 3) Enabling stage-specific therapeutic testing during the pre-aneurysmal window. By bridging molecular insights with clinical progression patterns, this strategic transition from reactive aneurysm repair to early intervention during the arteriomegaly phase provides innovative approaches to prevent progressive vascular damage.
